# Supplementary material for: Clinical and Diagnostic Utility of Genomic Profiling for Digestive Cancers: Real-World Evidence from Japan
Source: Cancers (Basel). 2024 Apr 15;16(8):1504. doi: 10.3390/cancers16081504 (PMC11048180; doi:10.3390/cancers16081504)
Supplement: Supplementary file 1 [file cancers-16-01504-s001.zip › Supplementary_Figures.pptx]

## Slide 1
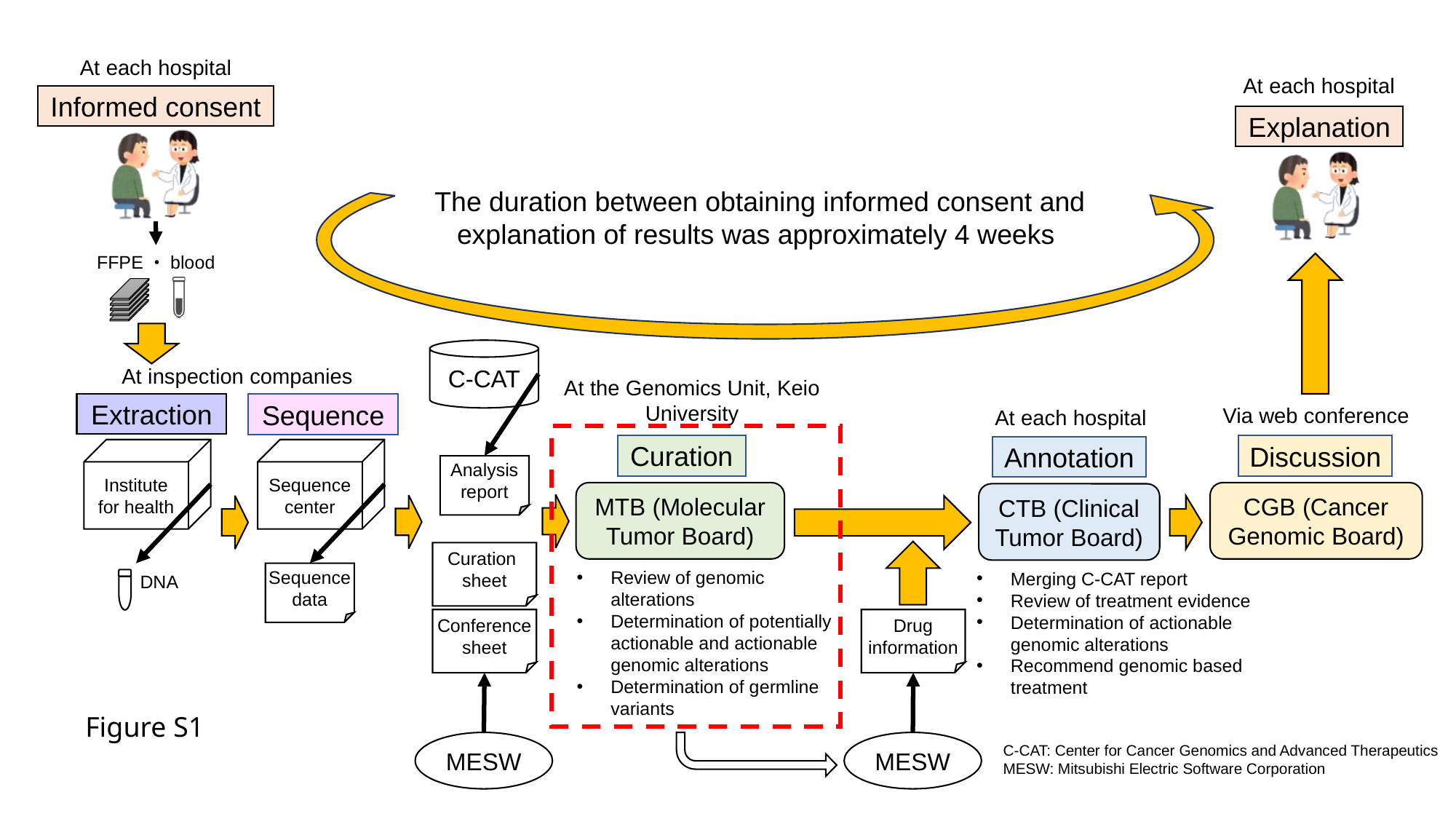

At each hospital
At each hospital
Informed consent
Explanation
The duration between obtaining informed consent and explanation of results was approximately 4 weeks
FFPE・blood
C-CAT
At inspection companies
At the Genomics Unit, Keio University
Extraction
Sequence
Via web conference
At each hospital
Curation
Discussion
Annotation
Institute for health
Sequence center
Analysis
report
MTB (Molecular
Tumor Board)
CGB (Cancer
Genomic Board)
CTB (Clinical
Tumor Board)
Curation
sheet
Review of genomic alterations
Determination of potentially actionable and actionable genomic alterations
Determination of germline variants
Merging C-CAT report
Review of treatment evidence
Determination of actionable genomic alterations
Recommend genomic based treatment
Sequence
data
DNA
Conference
sheet
Drug
information
Figure S1
MESW
MESW
C-CAT: Center for Cancer Genomics and Advanced Therapeutics
MESW: Mitsubishi Electric Software Corporation

## Slide 2
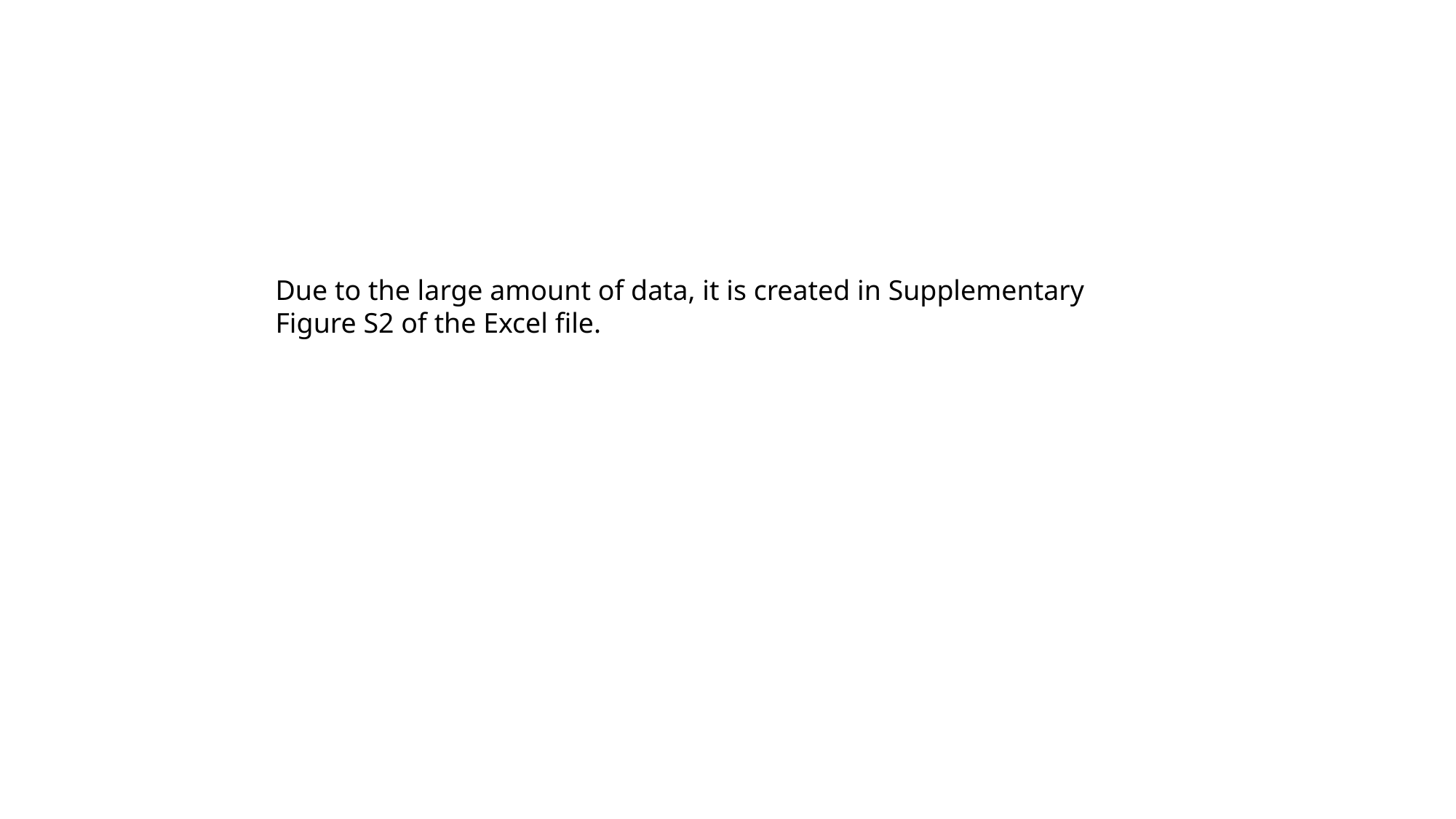

Due to the large amount of data, it is created in Supplementary Figure S2 of the Excel file.

## Slide 3
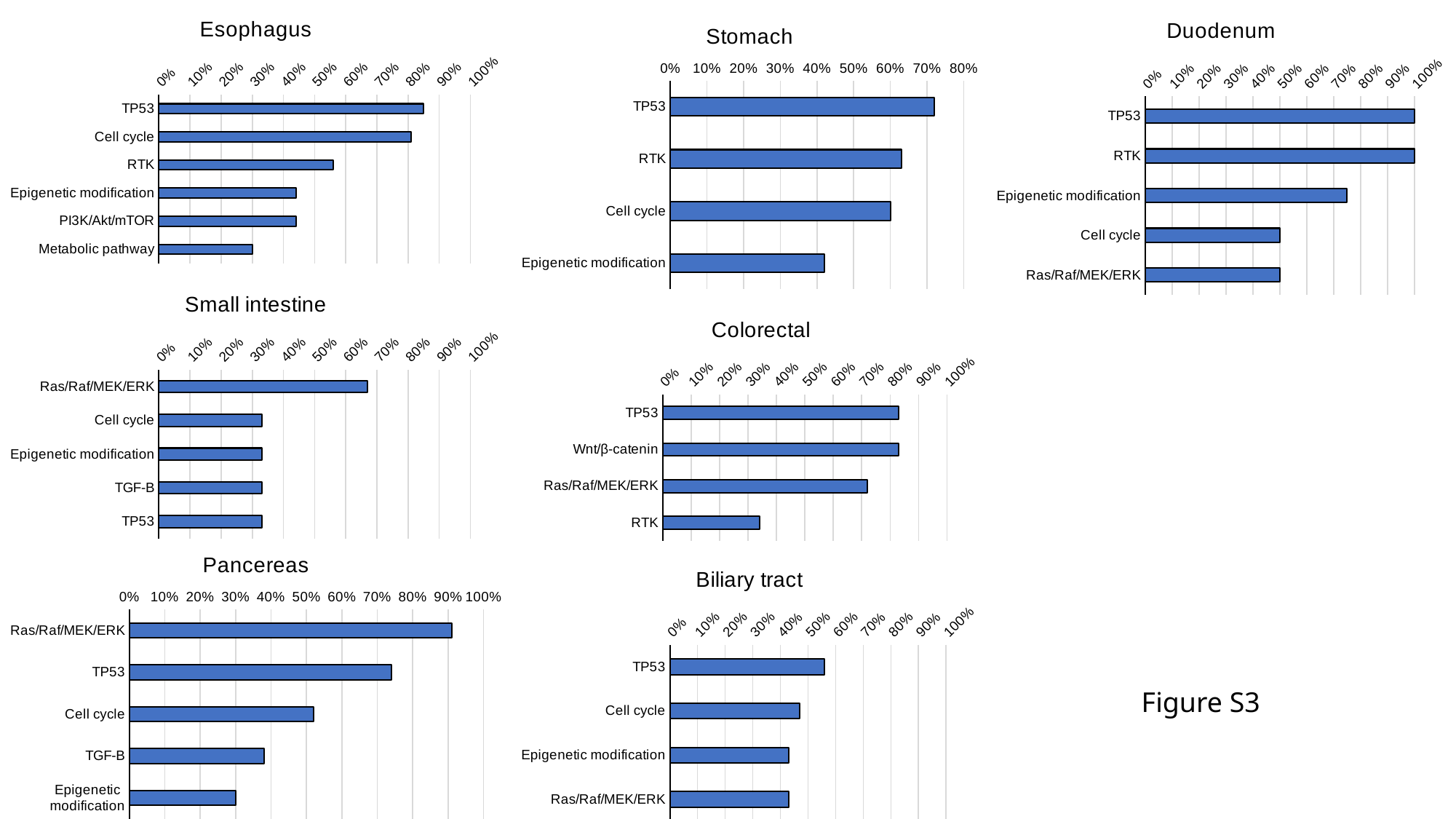

### Chart: Esophagus
| Category | |
|---|---|
| TP53 | 0.85 |
| Cell cycle | 0.81 |
| RTK | 0.56 |
| Epigenetic modification | 0.44 |
| PI3K/Akt/mTOR | 0.44 |
| Metabolic pathway | 0.3 |
### Chart: Duodenum
| Category | |
|---|---|
| TP53 | 1.0 |
| RTK | 1.0 |
| Epigenetic modification | 0.75 |
| Cell cycle | 0.5 |
| Ras/Raf/MEK/ERK | 0.5 |
### Chart: Stomach
| Category | |
|---|---|
| TP53 | 0.72 |
| RTK | 0.63 |
| Cell cycle | 0.6 |
| Epigenetic modification | 0.42 |
### Chart: Small intestine
| Category | |
|---|---|
| Ras/Raf/MEK/ERK | 0.67 |
| Cell cycle | 0.33 |
| Epigenetic modification | 0.33 |
| TGF-B | 0.33 |
| TP53 | 0.33 |
### Chart: Colorectal
| Category | |
|---|---|
| TP53 | 0.83 |
| Wnt/β-catenin | 0.83 |
| Ras/Raf/MEK/ERK | 0.72 |
| RTK | 0.34 |
### Chart: Pancereas
| Category | |
|---|---|
| Ras/Raf/MEK/ERK | 0.91 |
| TP53 | 0.74 |
| Cell cycle | 0.52 |
| TGF-B | 0.38 |
| Epigenetic modification | 0.3 |
### Chart: Biliary tract
| Category | |
|---|---|
| TP53 | 0.56 |
| Cell cycle | 0.47 |
| Epigenetic modification | 0.43 |
| Ras/Raf/MEK/ERK | 0.43 |Figure S3

## Slide 4
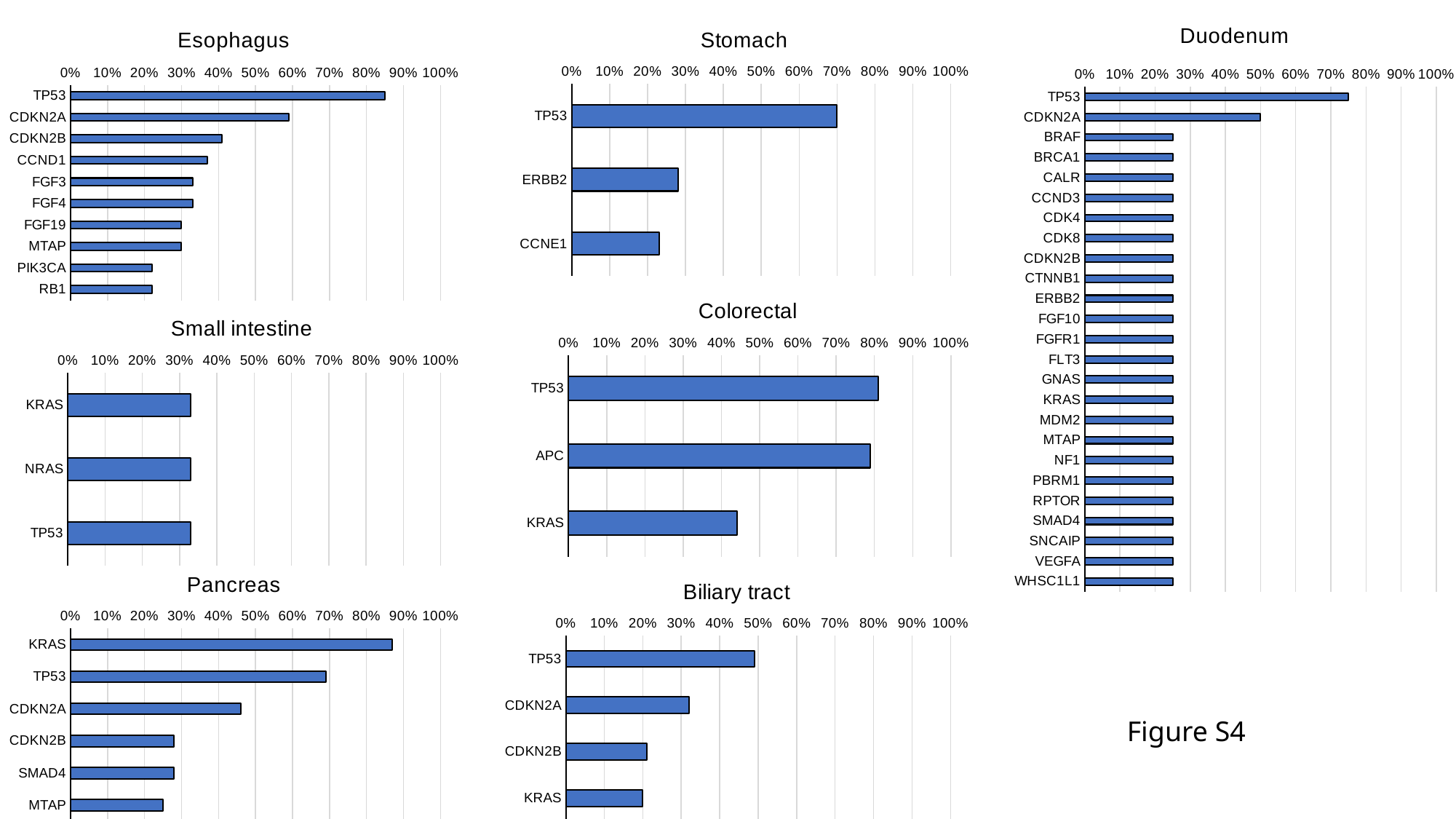

### Chart: Duodenum
| Category | |
|---|---|
| TP53 | 0.75 |
| CDKN2A | 0.5 |
| BRAF | 0.25 |
| BRCA1 | 0.25 |
| CALR | 0.25 |
| CCND3 | 0.25 |
| CDK4 | 0.25 |
| CDK8 | 0.25 |
| CDKN2B | 0.25 |
| CTNNB1 | 0.25 |
| ERBB2 | 0.25 |
| FGF10 | 0.25 |
| FGFR1 | 0.25 |
| FLT3 | 0.25 |
| GNAS | 0.25 |
| KRAS | 0.25 |
| MDM2 | 0.25 |
| MTAP | 0.25 |
| NF1 | 0.25 |
| PBRM1 | 0.25 |
| RPTOR | 0.25 |
| SMAD4 | 0.25 |
| SNCAIP | 0.25 |
| VEGFA | 0.25 |
| WHSC1L1 | 0.25 |
### Chart: Esophagus
| Category | |
|---|---|
| TP53 | 0.85 |
| CDKN2A | 0.59 |
| CDKN2B | 0.41 |
| CCND1 | 0.37 |
| FGF3 | 0.33 |
| FGF4 | 0.33 |
| FGF19 | 0.3 |
| MTAP | 0.3 |
| PIK3CA | 0.22 |
| RB1 | 0.22 |
### Chart: Stomach
| Category | |
|---|---|
| TP53 | 0.7 |
| ERBB2 | 0.28 |
| CCNE1 | 0.23 |
### Chart: Colorectal
| Category | |
|---|---|
| TP53 | 0.81 |
| APC | 0.79 |
| KRAS | 0.44 |
### Chart: Small intestine
| Category | |
|---|---|
| KRAS | 0.33 |
| NRAS | 0.33 |
| TP53 | 0.33 |
### Chart: Pancreas
| Category | |
|---|---|
| KRAS | 0.87 |
| TP53 | 0.69 |
| CDKN2A | 0.46 |
| CDKN2B | 0.28 |
| SMAD4 | 0.28 |
| MTAP | 0.25 |
### Chart: Biliary tract
| Category | |
|---|---|
| TP53 | 0.49 |
| CDKN2A | 0.32 |
| CDKN2B | 0.21 |
| KRAS | 0.2 |Figure S4

## Slide 5
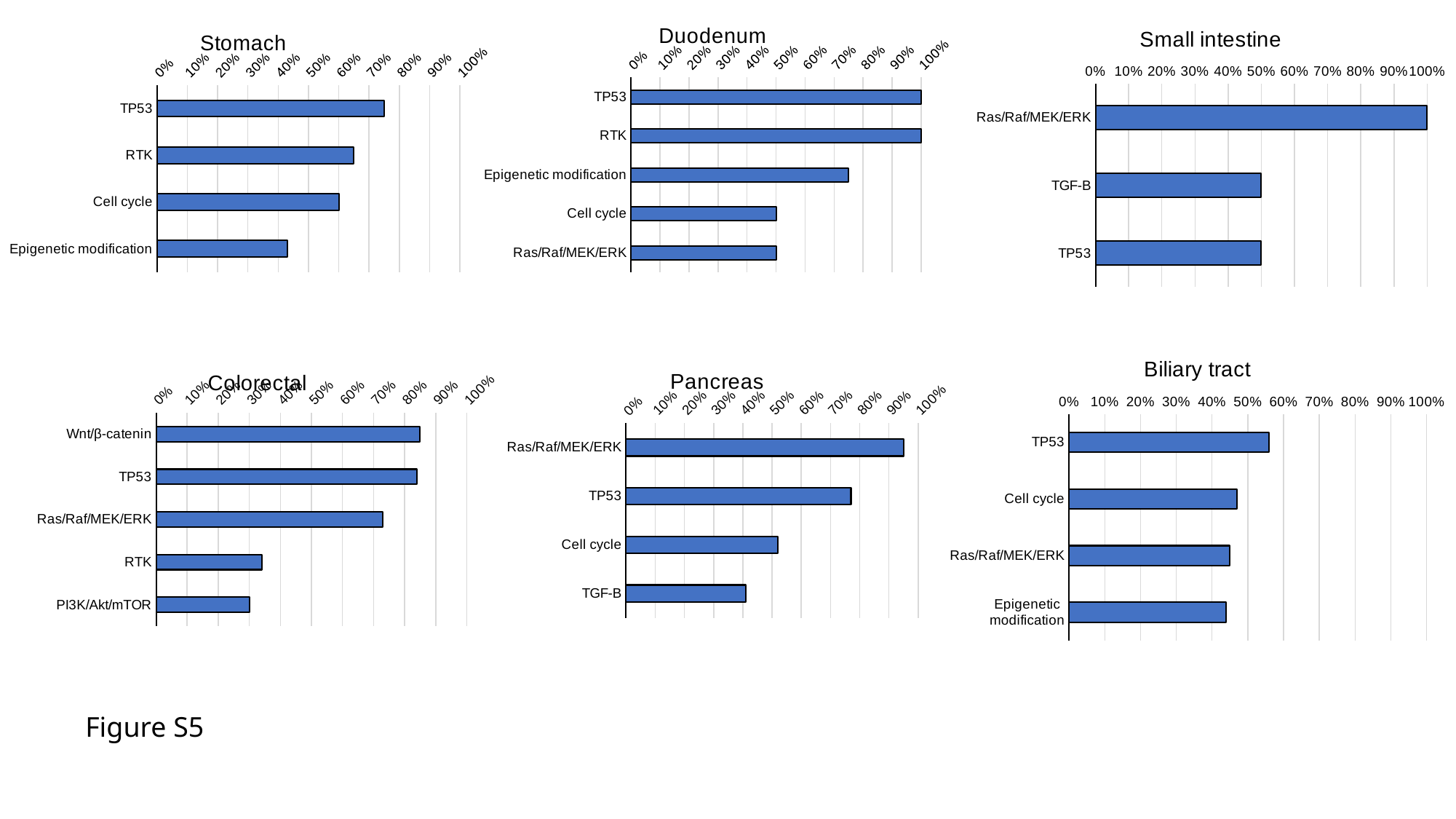

### Chart: Duodenum
| Category | |
|---|---|
| TP53 | 1.0 |
| RTK | 1.0 |
| Epigenetic modification | 0.75 |
| Cell cycle | 0.5 |
| Ras/Raf/MEK/ERK | 0.5 |
### Chart: Small intestine
| Category | |
|---|---|
| Ras/Raf/MEK/ERK | 1.0 |
| TGF-B | 0.5 |
| TP53 | 0.5 |
### Chart: Stomach
| Category | |
|---|---|
| TP53 | 0.75 |
| RTK | 0.65 |
| Cell cycle | 0.6 |
| Epigenetic modification | 0.43 |
### Chart: Biliary tract
| Category | |
|---|---|
| TP53 | 0.56 |
| Cell cycle | 0.47 |
| Ras/Raf/MEK/ERK | 0.45 |
| Epigenetic modification | 0.44 |
### Chart: Pancreas
| Category | |
|---|---|
| Ras/Raf/MEK/ERK | 0.95 |
| TP53 | 0.77 |
| Cell cycle | 0.52 |
| TGF-B | 0.41 |
### Chart: Colorectal
| Category | |
|---|---|
| Wnt/β-catenin | 0.85 |
| TP53 | 0.84 |
| Ras/Raf/MEK/ERK | 0.73 |
| RTK | 0.34 |
| PI3K/Akt/mTOR | 0.3 |Figure S5

## Slide 6
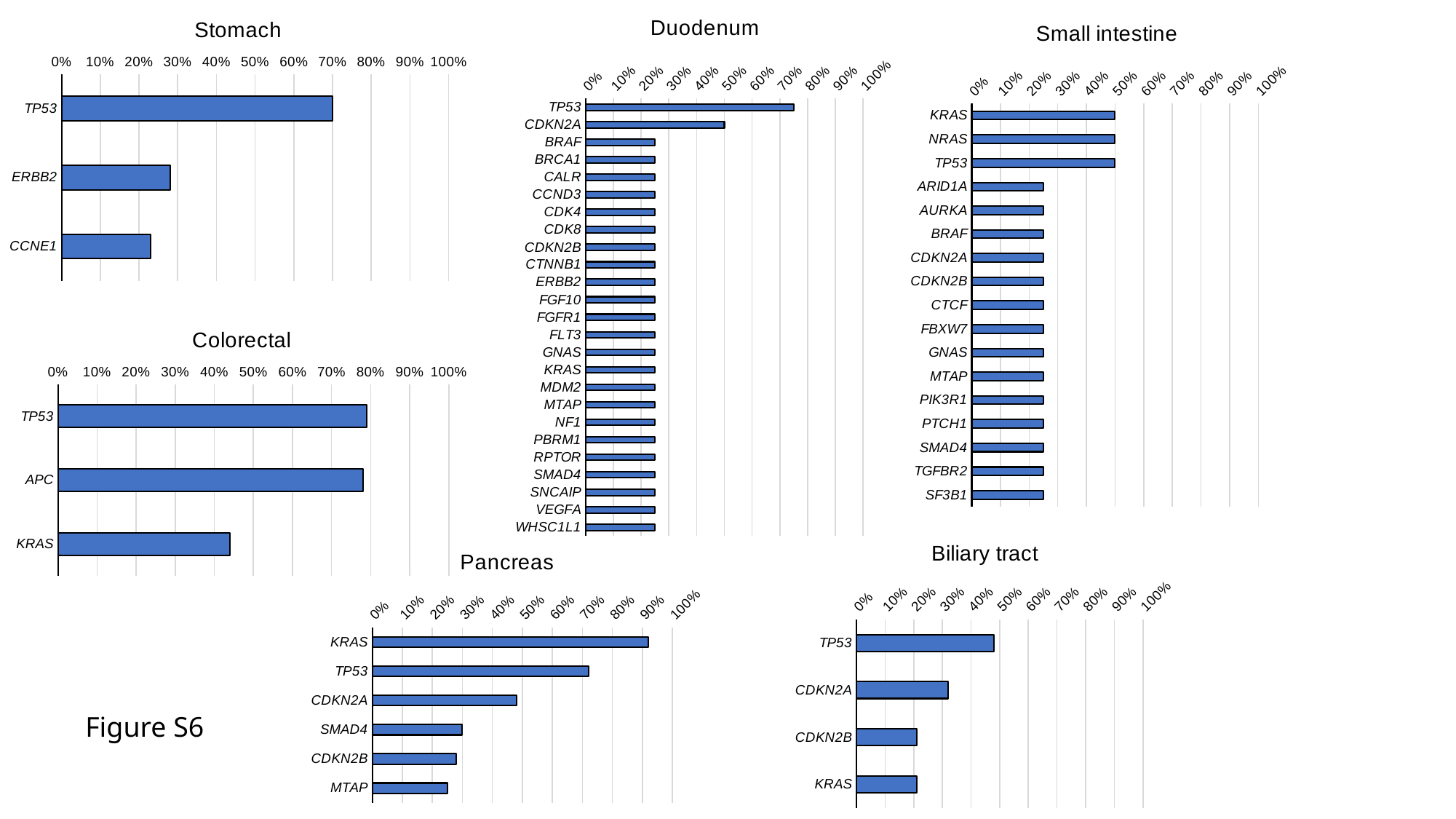

### Chart: Stomach
| Category | |
|---|---|
| TP53 | 0.7 |
| ERBB2 | 0.28 |
| CCNE1 | 0.23 |
### Chart: Duodenum
| Category | |
|---|---|
| TP53 | 0.75 |
| CDKN2A | 0.5 |
| BRAF | 0.25 |
| BRCA1 | 0.25 |
| CALR | 0.25 |
| CCND3 | 0.25 |
| CDK4 | 0.25 |
| CDK8 | 0.25 |
| CDKN2B | 0.25 |
| CTNNB1 | 0.25 |
| ERBB2 | 0.25 |
| FGF10 | 0.25 |
| FGFR1 | 0.25 |
| FLT3 | 0.25 |
| GNAS | 0.25 |
| KRAS | 0.25 |
| MDM2 | 0.25 |
| MTAP | 0.25 |
| NF1 | 0.25 |
| PBRM1 | 0.25 |
| RPTOR | 0.25 |
| SMAD4 | 0.25 |
| SNCAIP | 0.25 |
| VEGFA | 0.25 |
| WHSC1L1 | 0.25 |
### Chart: Small intestine
| Category | |
|---|---|
| KRAS | 0.5 |
| NRAS | 0.5 |
| TP53 | 0.5 |
| ARID1A | 0.25 |
| AURKA | 0.25 |
| BRAF | 0.25 |
| CDKN2A | 0.25 |
| CDKN2B | 0.25 |
| CTCF | 0.25 |
| FBXW7 | 0.25 |
| GNAS | 0.25 |
| MTAP | 0.25 |
| PIK3R1 | 0.25 |
| PTCH1 | 0.25 |
| SMAD4 | 0.25 |
| TGFBR2 | 0.25 |
| SF3B1 | 0.25 |
### Chart: Colorectal
| Category | |
|---|---|
| TP53 | 0.79 |
| APC | 0.78 |
| KRAS | 0.44 |
### Chart: Biliary tract
| Category | |
|---|---|
| TP53 | 0.48 |
| CDKN2A | 0.32 |
| CDKN2B | 0.21 |
| KRAS | 0.21 |
### Chart: Pancreas
| Category | |
|---|---|
| KRAS | 0.92 |
| TP53 | 0.72 |
| CDKN2A | 0.48 |
| SMAD4 | 0.3 |
| CDKN2B | 0.28 |
| MTAP | 0.25 |Figure S6

## Slide 7
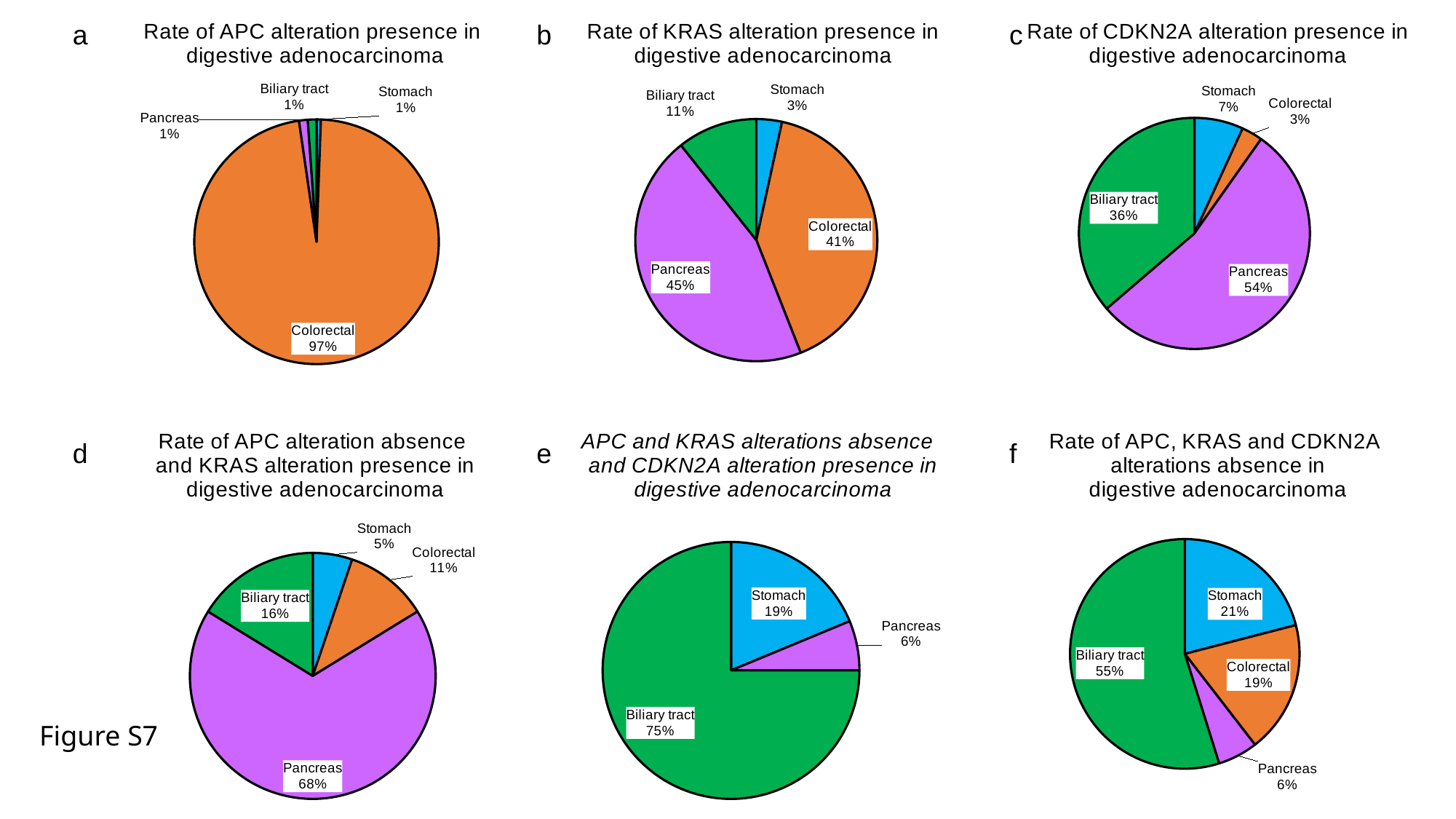

### Chart: Rate of APC alteration presence in
digestive adenocarcinoma
| Category | APC alteration |
|---|---|
| Stomach | 1.0 |
| Colorectal | 169.0 |
| Pancreas | 2.0 |
| Biliary tract | 2.0 |
### Chart: Rate of KRAS alteration presence in
digestive adenocarcinoma
| Category | KRAS alteration |
|---|---|
| Stomach | 8.0 |
| Colorectal | 95.0 |
| Pancreas | 106.0 |
| Biliary tract | 25.0 |
### Chart: Rate of CDKN2A alteration presence in
digestive adenocarcinoma
| Category | CDKN2A alteration |
|---|---|
| Stomach | 7.0 |
| Colorectal | 3.0 |
| Pancreas | 55.0 |
| Biliary tract | 37.0 |a
b
c
### Chart: Rate of APC alteration absence
and KRAS alteration presence in
digestive adenocarcinoma
| Category | APC alteration negative and KRAS alteration positive |
|---|---|
| Stomach | 8.0 |
| Colorectal | 17.0 |
| Pancreas | 104.0 |
| Biliary tract | 25.0 |
### Chart: APC and KRAS alterations absence
and CDKN2A alteration presence in
digestive adenocarcinoma
| Category | APC and KRAS alteration negative, and CDKN2A alteration positive |
|---|---|
| Stomach | 6.0 |
| Colorectal | 0.0 |
| Pancreas | 2.0 |
| Biliary tract | 24.0 |
### Chart: Rate of APC, KRAS and CDKN2A
alterations absence in
digestive adenocarcinoma
| Category | APC, KRAS and CDKN2A alterations negative |
|---|---|
| Stomach | 26.0 |
| Colorectal | 23.0 |
| Pancreas | 7.0 |
| Biliary tract | 68.0 |d
e
f
Figure S7

## Slide 8
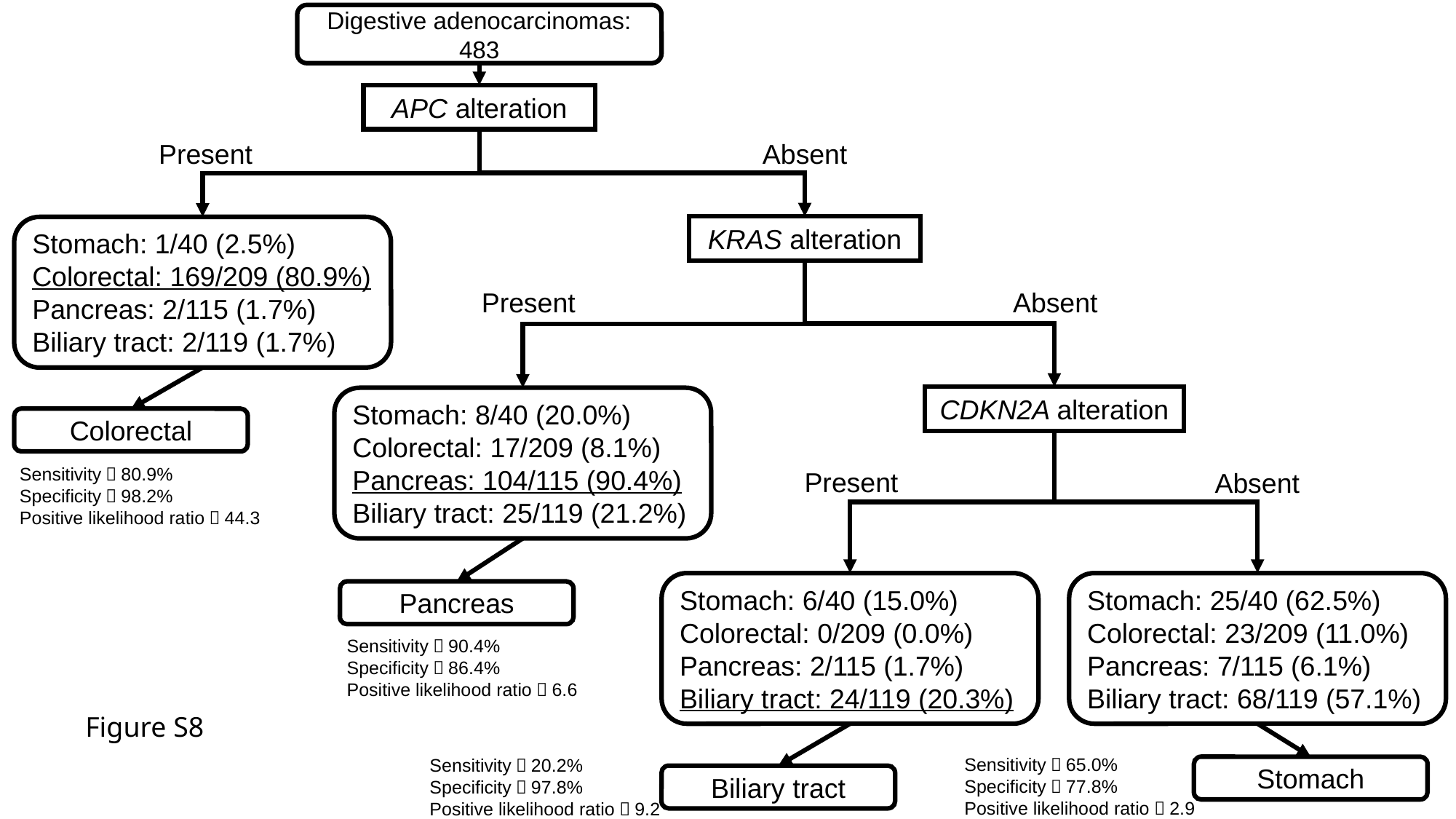

Digestive adenocarcinomas: 483
APC alteration
Present
Absent
KRAS alteration
Stomach: 1/40 (2.5%)
Colorectal: 169/209 (80.9%)
Pancreas: 2/115 (1.7%)
Biliary tract: 2/119 (1.7%)
Present
Absent
CDKN2A alteration
Stomach: 8/40 (20.0%)
Colorectal: 17/209 (8.1%)
Pancreas: 104/115 (90.4%)
Biliary tract: 25/119 (21.2%)
Colorectal
Sensitivity：80.9%
Specificity：98.2%
Positive likelihood ratio：44.3
Present
Absent
Stomach: 25/40 (62.5%)
Colorectal: 23/209 (11.0%)
Pancreas: 7/115 (6.1%)
Biliary tract: 68/119 (57.1%)
Stomach: 6/40 (15.0%)
Colorectal: 0/209 (0.0%)
Pancreas: 2/115 (1.7%)
Biliary tract: 24/119 (20.3%)
Pancreas
Sensitivity：90.4%
Specificity：86.4%
Positive likelihood ratio：6.6
Figure S8
Sensitivity：65.0%
Specificity：77.8%
Positive likelihood ratio：2.9
Sensitivity：20.2%
Specificity：97.8%
Positive likelihood ratio：9.2
Stomach
Biliary tract
